# Impact of older donor age in kidney transplants in a biopsy-based observational study

**DOI:** 10.1172/jci.insight.199060

**Published:** 2026-05-07

**Authors:** Katelynn Madill-Thomsen, Martina Mackova, Jessica Chang, Enver Akalin, Tarek Alhamad, Sanjiv Anand, Miha Arnol, Rajendra Baliga, Mirosław Banasik, Christopher Blosser, Georg Böhmig, Daniel Brennan, Jonathan Bromberg, Klemens Budde, Andrzej Chamienia, Kevin V. Chow, Michał Ciszek, Declan de Freitas, Dominika Dęborska-Materkowska, Alicja Dębska-Ślizień, Arjang Djamali, Leszek Domański, Magdalena Durlik, Gunilla Einecke, Farsad Eskandary, Richard Fatica, Iman Francis, Justyna Fryc, John Gill, Jagbir Gill, Maciej Glyda, Sita Gourishankar, Marta Gryczman, Gaurav Gupta, Petra Hruba, Peter Hughes, Arksarapuk Jittirat, Zeljka Jurekovic, Layla Kamal, Mahmoud Kamel, Sam Kant, Nika Kojc, Joanna Konopa, James Lan, Roslyn Mannon, Arthur Matas, Joanna Mazurkiewicz, Marius Miglinas, Thomas Mueller, Marek Myślak, Beata Naumnik, Anita Patel, Agnieszka Perkowska-Ptasińska, Michael Picton, Grzegorz Piecha, Emillio Poggio, Silvie Rajnochova Bloudickova, Thomas Schachtner, Sung Shin, Soroush Shojai, Majid Sikosana, Janka Slatinská, Katarzyna Smykal-Jankowiak, Ashish Solanki, Zeljka Veceric Haler, Ondrej Viklicky, Ksenija Vucur Simic, Matthew R. Weir, Andrzej Wiecek, Zbigniew Włodarczyk, Ziad Zaky, Philip F. Halloran

**Affiliations:** 1 Alberta Transplant Applied Genomics Centre, Edmonton, Alberta, Canada.; 2 Montefiore Medical Center, Bronx, New York, USA.; 3 Washington University at St. Louis, St. Louis, Missouri, USA.; 4 Intermountain Transplant Services, Murray, Utah, USA.; 5 University of Ljubljana, Ljubljana, Slovenia.; 6 Tampa General Hospital, Tampa, Florida, USA.; 7 Medical University of Wrocław, Wrocław. Poland.; 8 University of Washington, Seattle, Washington, USA.; 9 Medical University of Vienna, Vienna, Austria.; 10 Johns Hopkins University School of Medicine, Baltimore, Maryland, USA.; 11 University of Maryland, Baltimore, Maryland, USA.; 12 Charite-Medical University of Berlin, Berlin, Germany.; 13 Medical University of Gdańsk, Gdańsk, Poland.; 14 The Royal Melbourne Hospital, Parkville, Australia.; 15 Warsaw Medical University, Warsaw, Poland.; 16 Manchester Royal Infirmary, Manchester, United Kingdom.; 17 University of Wisconsin, Madison, Wisconsin, USA.; 18 Pomeranian Medical University, Szczecin, Poland.; 19 Medical University of Hannover, Hannover, Germany.; 20 Cleveland Clinic Foundation, Cleveland, Ohio, USA.; 21 Henry Ford Transplant Institute, Detroit, Michigan, USA.; 22 Medical University in Bialystok, Białystok, Poland.; 23 St. Paul’s Hospital, Vancouver, British Columbia, Canada.; 24 Wojewodzki Hospital, Poznan, Poland.; 25 University of Alberta, Edmonton, Alberta, Canada.; 26 Virginia Commonwealth University, Richmond, Virginia, USA.; 27 Institute for Experimental and Clinical Medicine, Prague, Czech Republic.; 28 University Hospital Cleveland Medical Center, Cleveland, Ohio, USA.; 29 University Hospital Merkur, Zagreb, Croatia.; 30 University of Ljubljana, Ljubljana, Slovenia.; 31 University of Alabama at Birmingham, Birmingham, Alabama, USA.; 32 University on Minnesota, Minneapolis, Minnesota, USA.; 33 Vilnius University Hospital Santaros Klinikos, Vilnius, Lithuania.; 34 University Hospital Zurich, Zurich, Switzerland.; 35 Silesian Medical University, Katowice, Poland.; 36 University of Ulsan College of Medicine/Asan Medical Center, Seoul, South Korea.; 37 University Hospital no. 1, Bydgoszcz, Poland.

**Keywords:** Clinical Research, Nephrology, Organ transplantation

## Abstract

Because older donor age is a major concern when considering kidneys for potential transplantation, we explored the actual effect of donor age on the features of kidneys that have been transplanted. We studied the correlations of donor age with molecular injury and rejection scores in 4,502 kidney transplant biopsies assessed by microarrays as well as function and postbiopsy survival. We used multivariable analyses to correct for the correlations of donor age with other predictive variables: recipient age, time of biopsy after transplant, and deceased versus living donors. Older donor age correlated with lower glomerular filtration rate (GFR) and increased acute and chronic injury transcripts but had no effect on rejection, which was anticorrelated with recipient age. Acute injury transcripts peaked immediately after transplant and regressed. Older donor age had little effect on acute molecular injury immediately after transplant but strongly increased molecular injury scores at later times, peaking about 1-year after transplant, indicating that older age does not increase molecular injury but increases failed repair after injury. As expected, older donor age correlated with increased chronic injury and lower GFR, evident from the earliest time after transplant, pretransplant aging. However, despite substantial age-related effects, the quantitative contribution of donor aging to molecular injury, function, and survival was very small.

## Abstract 2

In kidney transplants, older donor age correlates with more injury and lower function but the actual impact of age on function and survival is small.

## Introduction

Donor age is a major concern in organ transplantation because of the potential that age-associated parenchymal deterioration will reduce function and impair survival. In kidney transplantation in the United States, this concern contributes to the discarding of more than 5,000 potentially transplantable kidneys, many from older donors, despite more than 100,000 people being on the waiting list (1–3). Such kidneys are often successfully transplanted in other countries (4). Donor age is an element in the definition of extended criteria donors and is a major driver of kidney risk indices (5–7). Many have advocated for more transplantation of kidneys from older donors (8), but fear of adverse outcomes continues to drive discards (9, 10), and difficulty placing such kidneys causes longer cold ischemic times. Donor age also contributes to the discarding of many hearts and lungs available for transplantation.

The background for this problem is the incomplete understanding of the actual molecular consequences of older kidney age in kidneys (10). Biological age of a tissue is difficult to define (11), and the actual effects of aging in transplanted organs are multifactorial (12) and nonlinear (13) and are the subject of increasing public interest in aging “clocks” (14). Aging obviously affects many biological processes, e.g., telomere shortening and somatic cell senescence (15). A recent proteomic and histological analysis of 516 samples from 13 human tissues spanning 5 decades (not including kidney) found an inflection in age effects around age 50 years, with widespread transcriptome-proteome decoupling in older organs (16). (Because of this, we have used age 50 years as a cutoff in some of the studies presented here.) In native kidneys, the effects on estimated glomerular filtration rate (eGFR) have been thoroughly explored in longitudinal studies (17, 18), and the histologic and molecular effects of kidney aging have been extensively studied (17, 19, 20), including relationships to chronic kidney disease (CKD) (21). Cellular deconvolution studies using single kidney cell transcriptomes found age-related increases in the expression of genes representing B and T cells, macrophages, myofibroblasts, and fibroblasts (22), reflecting parenchymal changes, mild atrophy-fibrosis (nephron loss), and inflammation. Whether these inflammation and senescence changes affect the recipient alloimmune response — either positively or negatively — is not clear, particularly since donor age and recipient age tend to be correlated.

A new issue relevant to the problem of older kidney age in transplants is that aging increases the expression of molecular acute kidney injury–induced (AKI-induced) genes and genes related to failed repair of injury (23). The processes of donation and implantation induce expression of AKI transcripts like injury-induced genes (IRRATs), annotated in early human kidney transplant biopsies, and tissue injury and repair–associated transcripts (IRITD3) annotated in mouse kidney isografts (24, 25). Single-nucleus transcriptome studies show that some injured cells fail to repair, causing persistent expression of transcripts related to acute injury long after the majority of the AKI-induced changes have resolved (26–30). Failed repair cells share features with senescent cells (30), and some gene sets such as TAL_New4 (23, 30, 31) correlate with epithelial-mesenchymal transition (EMT) (25, 30). Transcript sets annotated as AKI-induced and those annotated in failed repair cells share a similar time course in early transplant biopsies, peaking immediately after transplant and regressing in the following weeks, but continuing to be detectable in biopsies at later times, where they correlate with depressed eGFR and increased probability of failure (32–35).

The interpretation of donor age effects in transplant biopsies is complicated by the association of donor age with other variables such as recipient age, time of biopsy after transplant (TxBx), and donor type (deceased donors vs. living donors [DDvLD]). The present study aimed to use multivariable analyses to define and quantify the effects of older donor age on injury, rejection, eGFR, and outcomes in kidney transplants undergoing biopsies, controlling for other key variables. We focused on donor age at transplantation because this is the time of critical decisions and included TxBx to account for aging and other factors after transplant. Although centers use many other criteria for evaluating donor quality, many of the kidneys in this cohort are from donors aged <50 years, reflecting concerns about transplanting kidneys from older donors. We used multivariable adaptive regression splines (MARS) analysis to adjust for confounders in the relationship of donor age to recipient age, DDvLD, and TxBx. We hypothesized that the actual quantitative effect of older donor age in transplanted kidneys, while significant, may be smaller than widely believed, a consideration that could be relevant to public policy.

## Results

### Study population.

**Figure 1:**
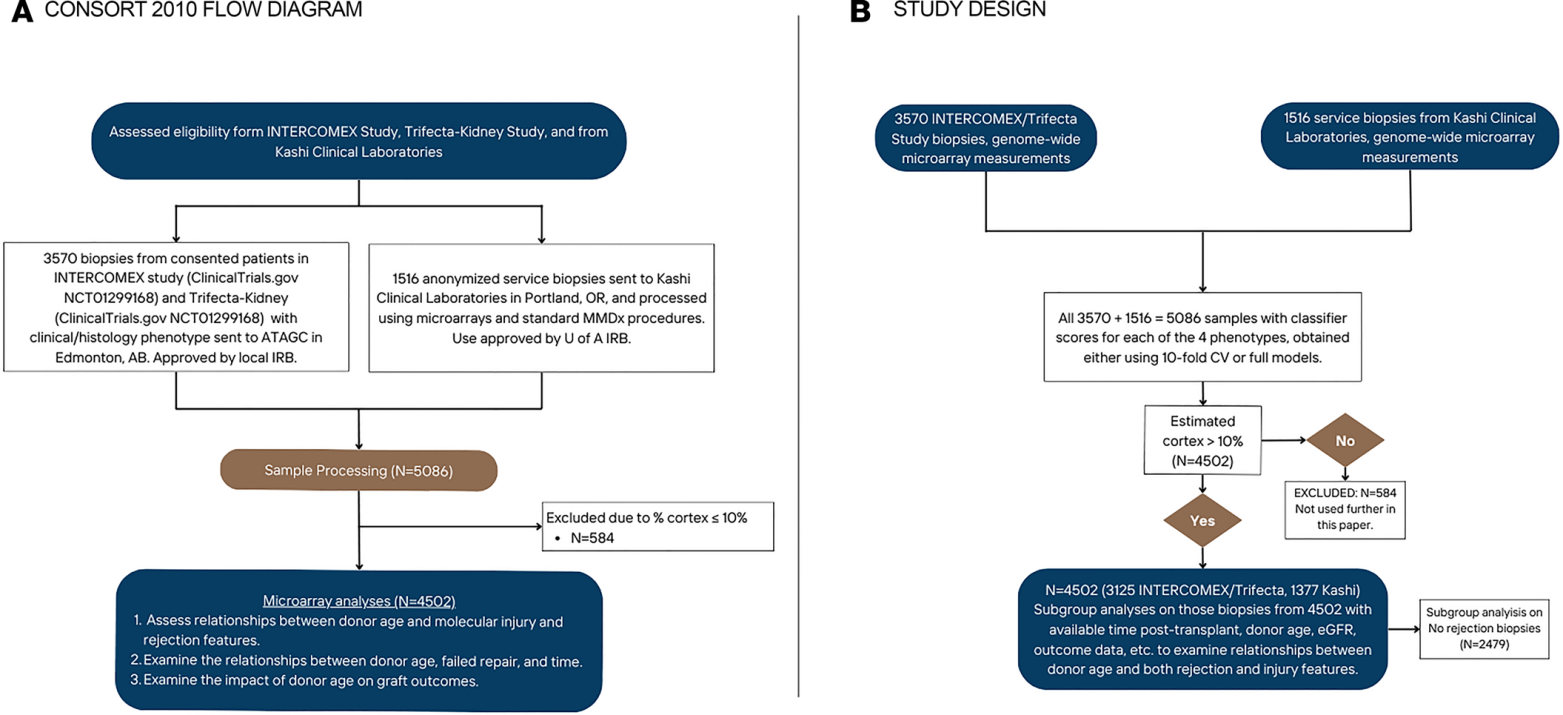
Consort diagram and study design for these analyses presented in the 4502 population. (**A**) CONSORT diagram showing biopsy inclusion for the INTERCOMEX and Trifecta-Kidney study (*N* = 3,570) dataset. (**B**) Study design.

The CONSORT diagram and study design are shown in Figure 1. Demographics for this study population of 4,502 biopsies from 3,611 patients are shown in Supplemental Table 1, as previously published (23, 36). Mean donor age was 43 years (671 donors were ≥50 years; mean, 59 years), and mean recipient age was 50 years; mean TxBx was 1,182 days or 3.2 years (median, 367 days).

As described in the Methods, we visualized the distribution of the 4,502 biopsies in the injury principal component analysis (PCA) model (Supplemental Figure 1, A and B; supplemental material available online with this article; https://doi.org/10.1172/jci.insight.199060DS1). We calculated injury PC1 (all injury), injury PC2 (early vs. late), and injury PC3 (epithelial remodeling), and assigned each biopsy to an injury archetype group: normal, AKI1, AKI2 (typical AKI), mild CKD, and CKDAKI (CKD with AKI transcripts) (37). We also visualized the rejection PCA model in Supplemental Figure 1, C and D.

### Relationship of donor age to other predictive variables in the biopsy population.

We studied the relationship of donor age to recipient age at transplant, donor type (DDvLD), and TxBx. An additional feature — biopsy for indication versus protocol — did not emerge as predictive, presumably because 82% of biopsies were for indications, and this variable was dropped from the analyses.

**Figure 2:**
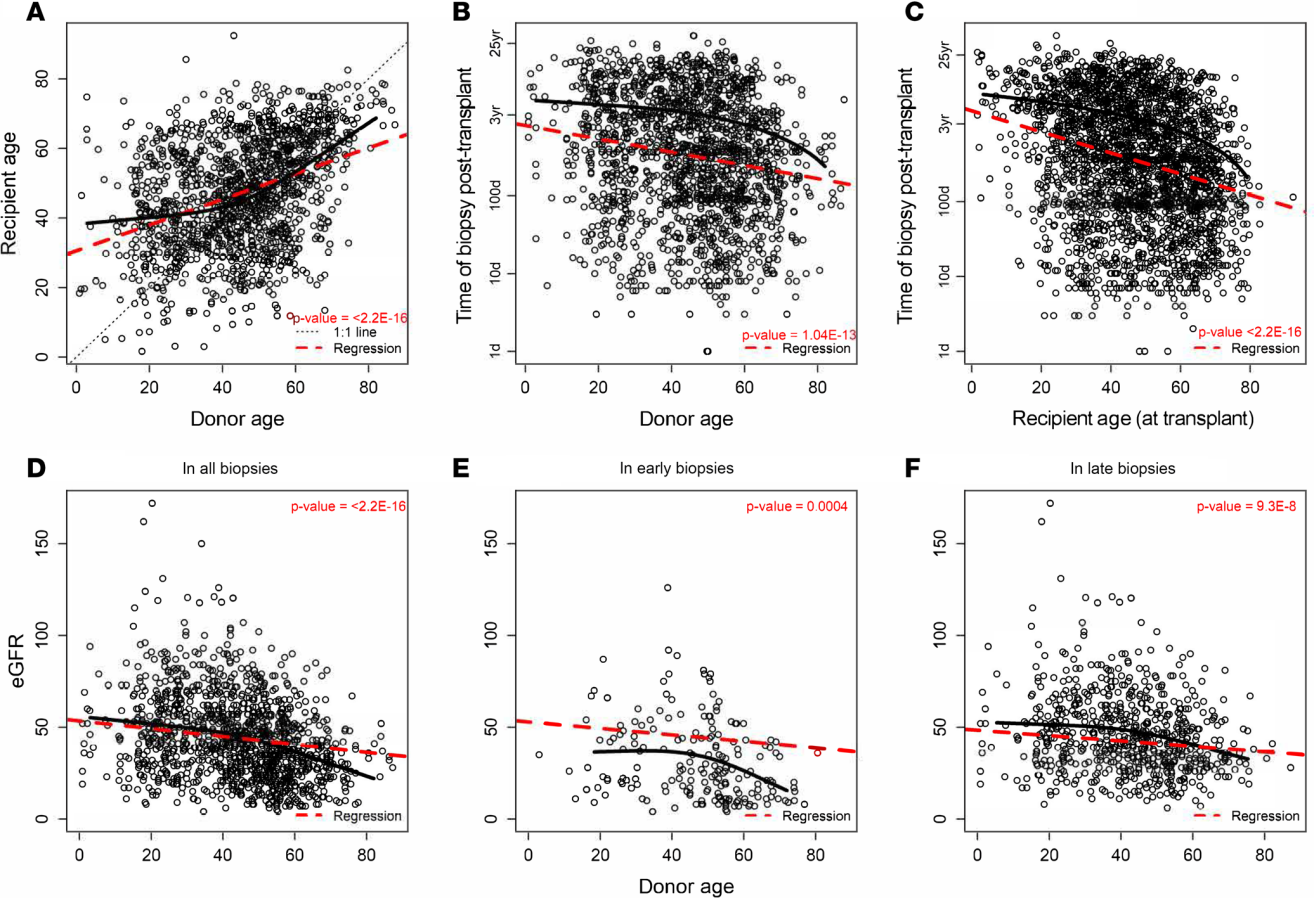
Scatterplot of time of biopsy after transplant, donor age, and recipient age in biopsies with available data. (**A**) Recipient age versus donor age (*N* = 1,802). (**B**) time of biopsy after transplant versus donor age (*N* = 1,802). (**C**) time of biopsy after transplant versus recipient age (*N* = 2,669). (**D**) eGFR versus donor age in all biopsies with available data for eGFR, time after transplant, and donor age (*N* = 2,669). (**E**) eGFR versus donor age in the early (≤42 days) biopsies (*N* = 270 of 2,669 from **C** and **D**). (**F**) eGFR versus donor age in the late (>1 year) biopsies (*N* = 911 of 2,669 from **C** and **D**). All *P* values reported on figure are for the regressions.

Donor age correlated with recipient age (Figure 2A, ρ = 0.38, *P* < 1.04 × 10^–16^), reflecting allocation policies that give young kidneys to young recipients and thus allocate older kidneys to older recipients.

TxBx correlated negatively with both donor age (Figure 2B, ρ = –0.19, *P* = 2.05 × 10^–13^) and recipient age (Figure 2C, ρ = –0.25, *P* < 2.2 × 10^–16^), indicating that transplants from older donors and/or recipients undergo more biopsies in the first weeks after transplant.

As expected, donor age was strongly anticorrelated with eGFR in all biopsies, in early biopsies (≤42 days), and in late biopsies (>1 year, Figure 2, D–F).

We assessed the effect of deceased donation (DD) versus living donation (LD) on TxBx, eGFR, and molecular injury and rejection (Table 1). (There were too few transplants recorded as being donation after cardiac death to analyze separately.) Compared with LD, DD kidneys were slightly later, had lower eGFR, had more positive injury PC3/epithelial remodeling, and had increased molecular injury: IRRATs, TAL_New4-failed repair, and injury PC1. However, DD kidneys did differ from LD in molecular rejection (here represented as rejection PC1).

### Multivariate adaptive regression splines analysis.

The strong relationship of donor age to the other predictive variables (recipient age, donor type, and TxBx) required the use of multivariate adaptive regression splines (MARS) analysis. This defined the relative association of each variable with the measurements at the time of biopsy — eGFR, molecular injury, and molecular rejection — holding the other predictive variables constant. (In some cases, interactions between variables were significant but this had little effect.)

Table 2 shows the relative importance in the cross-validated model of each predictive variable — TxBx, donor age, DDvLD, and recipient age — for predicting eGFR, IRRATs, injury PC1/2/3, and rejection PC1. Donor age was the only variable important in predicting eGFR. Donor age, TxBx, and DDvLD were all important for predicting expression of IRRATs and were also important for predicting injury PC1. TxBx was the only predictor of injury PC2. However, donor age was the major predictor of positive injury PC3. On the other hand, donor age had no positive or negative effect on rejection PC1, which anticorrelated with recipient age as expected.

**Figure 3:**
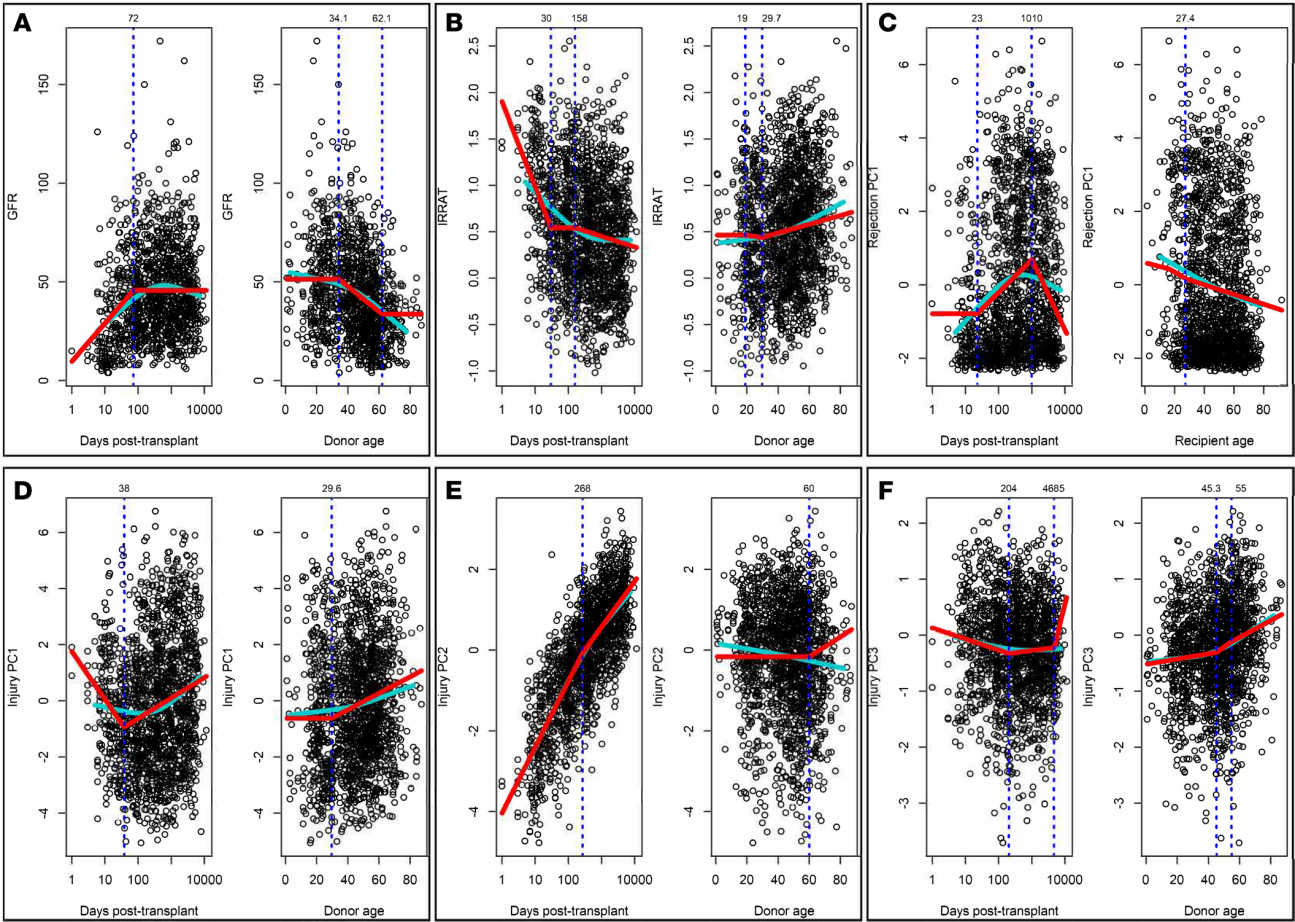
Partial dependence plots of multivariate adaptive regression spline models predicting clinical and injury-related variables, with log(days after transplant) and donor age as predictors. *N* = 1,802 samples with complete data. Predicted variables include (**A**) estimated glomerular filtration rate (GFR), (**B**) IRRATs, (**C**) rejection principal component 1 (PC1), (**D**) injury PC1, (**E**) injury principal component 2 (PC2), and (**F**) injury principal component 3 (PC3). Points represent individual biopsies. Red lines denote the fitted multivariate adaptive regression spline (MARS) partial dependence segments. Teal lines represent restricted cubic splines (3 knots). Blue dashed vertical lines indicate segment boundaries selected by MARS, with corresponding values shown above each panel. Predictor variables used in the models were log_10_(time after transplant), donor age, recipient age, and living versus deceased donors (0/1).

Having established the major associations in the cross-validated model, we used the full model in MARS analysis to visualize the shape of the relationships between predictive variables and the injury or rejection features of the kidneys (Figure 3). The red lines indicate the relationship between the ***x*** and ***y*** variables when all other predictor variables are held constant at their mean values using MARS analysis. The teal lines indicate simple univariate restricted cubic splines, shown for comparison, which generally agreed with MARS analyses.

In Figure 3A, eGFR was initially low, reflecting peritransplant injury, and recovered to a plateau around day 72. Donor age >30 years was associated with decreased eGFR, as expected.

In Figure 3B, the IRRAT score (AKI transcripts) (24) was highest early, reflecting peritransplant injury, and fell until plateauing at day 30, echoing eGFR trends. Donor age >30 years was associated with increased IRRAT scores. The TAL_New4-failed repair transcript scores performed very similarly to the IRRAT scores (Supplemental Figure 2), being highest early and falling until day 39; donor age >30 increased the failed repair transcript scores. Thus, as previously reported (23), IRRATs and TAL_New4 performed similarly, despite annotation in different systems, being strongly induced in early transplant biopsies before plateauing after about 6 weeks.

Because there was no positive or negative association of donor age with rejection, we examined the association of recipient age with rejection PC1 (Figure 3C). Rejection PC1 correlated strongly with TxBx, as in previous studies (36), peaking at around 1,000 days. Rejection PC1 declined steadily with recipient age.

The time course of injury PC1 was V-shaped (Figure 3D), higher in the earliest biopsies reflecting peritransplant injury, declining to a minimum at day 38, and then rising again in late biopsies, compatible with CKD changes.

The injury PC2 score correlated strongly with TxBx (Figure 3E), consistent with Table 2. Donor age had little correlation with injury PC2.

The injury PC3 score was less affected by time, although slightly higher early after transplant (Figure 3F). However, older donor age strongly increased injury PC3, particularly after age 60 years.

### Correlation of donor age with specific injury scores after time correction.

We used linear regression to explore the correlation of donor age with injury archetype scores and selected scores representing CKD changes after correcting for time (Table 3). Donor age was strongly positively correlated with the CKDAKI and AKI1 archetype scores and correlated negatively with the mild CKD, AKI2, and normal scores (Table 3).

Donor age was positively correlated with various CKD scores, including both the probability of fibrosis (ci>1_Prob_) and probability of histologic atrophy (ct>1_Prob_) classifier scores. Donor age was strongly correlated with expression of *CXCL6*, a chemokine that we previously found to be associated with CKDAKI and EMT (Table 3) but not AKI (23, 38) and strongly correlated with the CKDAKI score (ρ = 0.75) (23, 38).

### Correlation of recipient age with specific rejection scores after time correction.

We assessed the correlation of recipient age with rejection archetype scores (Supplemental Table 2) in a time-correlated model. Recipient age had a strong anticorrelation with rejection archetype scores, specifically T cell–mediated rejection 1 (TCMR1), TCMR2, and fully developed antibody-mediated rejection (FABMR) scores and correlated positively with the No Rejection score (Supplemental Table 2).

### Defining the time at which donor age effects operate.

**Figure 4:**
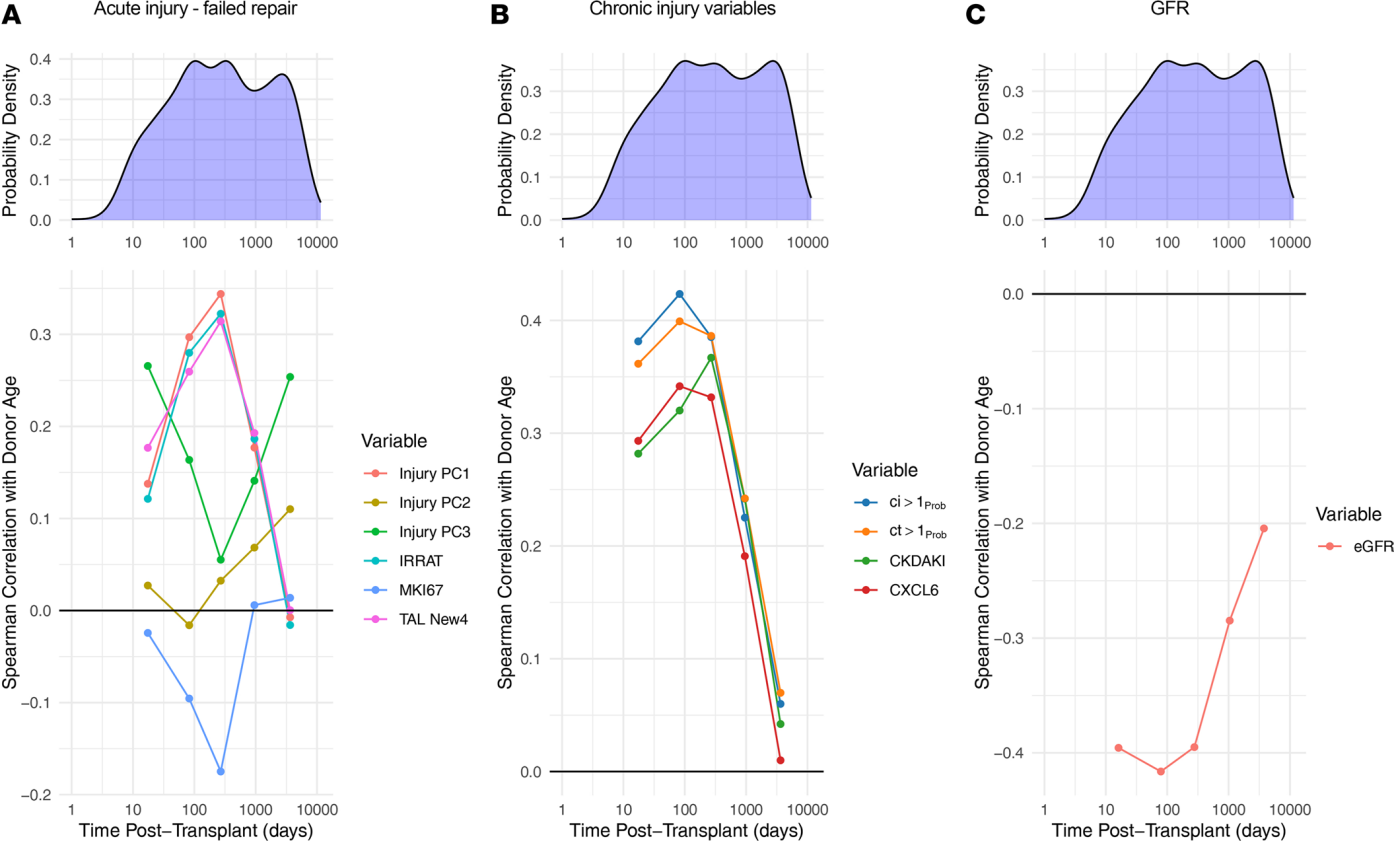
Changes in Spearman correlations over time between donor age and injury-related variables in biopsies classified as no rejection by our archetypal analysis. (**A**) The sample distribution’s probability density and correlation between donor age and injury PC scores, IRRATs, MKI67, and TAL_New4 (*N* = 1,021). (**B**) Sample distribution and correlation between donor age and ci>1_Prob_, ct>1_Prob_, CKDAKI, and CXCL6 (*N* = 1,021). (**C**) Sample distribution and correlation between donor age and eGFR (*N* = 767).

We visualized the strength of the donor age correlations with AKI-related scores, CKD-related scores, and eGFR at various times after transplant in Figure 4.

In Figure 4A, older donor age correlated with increased injury/failed-repair IRRAT and TAL_New4 genes most strongly at the intermediate times, peaking around 100 days, with lower correlations immediately after transplant and minimal correlations at late times. This was unexpected, given that the highest expression of IRRAT and TAL_New4 was immediately after transplant in Figure 3 and Supplemental Figure 2. Donor age also correlated with injury PC1 most strongly in the intermediate interval, like the IRRAT and TAL_New4 scores. Thus, the effect of older donor age is not on the early induction of molecular injury but much later, compatible with failed repair.

In contrast, donor age correlated strongly with injury PC3 in the early and late periods but much less at intermediate times. As in the MARS results, donor age had little effect on injury PC2.

Mitosis is an early transient event in acute injury (39) and correlates with negative injury PC2 in transplant biopsies (23). There was little relationship of donor age with expression of mitosis-associated gene *MKI67*, aside from weak anticorrelation at intermediate times.

Figure 4B explores the effect of time after transplant on the correlations of donor age with 4 chronic injury features: the 2 atrophy-fibrosis classifiers (ci>1Prob and ct>1Prob), the CKDAKI score, and chemokine *CXCL6*. All showed strong correlations at early and intermediate times after transplant that weakened at later times. The high correlation of donor age with chronicity features immediately after transplant suggests that these chronicity changes reflect aging in the donor kidney before transplantation, whereas the chronicity features at later times are mainly affected by rejection and other stresses, independent of donor age.

In Figure 4C, older donor age correlated negatively with eGFR immediately after transplant and through the first year and weakened at later times, similar to the correlations with chronicity features.

Supplemental Table 3 explores details of the correlations of donor age with various molecular injury measurements in 3 distinct time periods: early (≤42 days), intermediate (>42 days to ≤1 year), and late (>1 year). These results in the whole population were similar to results in biopsies with minimal rejection (Supplemental Table 4). As in Figure 4, donor age correlated with chronicity features early but with AKI/failed repair features at intermediate times.

In summary, older donor age correlated most strongly with AKI/failed repair genes at intermediate times after transplant, not at earlier times after transplant when expression of these genes was the highest, suggesting that older donor age increases failed repair of the injuries sustained in donation and transplantation. Older donor age correlated with chronicity features and low eGFR at early and intermediate times, suggesting pretransplant aging in the donor. Donor age had minimal correlations in late biopsies, despite the rising chronic injury scores in these biopsies.

### Genes and gene set enrichment analysis terms correlating with donor age.

Table 4 shows the top 20 genes (by *P* value) increased with donor age. Where annotated in our previous studies, some genes were noted to be injury-induced (potentially representing failed repair), e.g., *AKAP12* (*P* = 2.8E-23). Other donor-age correlated genes included the negative regulator of growth *CDKN1A* and cell junction gene protocadherins *PCDHA1* and *PCDHB2*, both of which correlated with injury PC3.

In gene set enrichment analysis (GSEA), the top pathways and gene sets in the genes positively enriched in biopsies with donor age ≥50 versus <50 years were dominated by the acute injury/failed repair gene sets (e.g., TAL_New4, IRITD3, and IRRATs) and EMT (Table 5).

### Quantifying the contributions of donor age to injury PCA, eGFR, and 3-year survival.

Having demonstrated the strong statistical relationship of donor age with failed repair and chronicity scores, we measured the actual net contribution of donor age to injury in the population.

**Figure 5:**
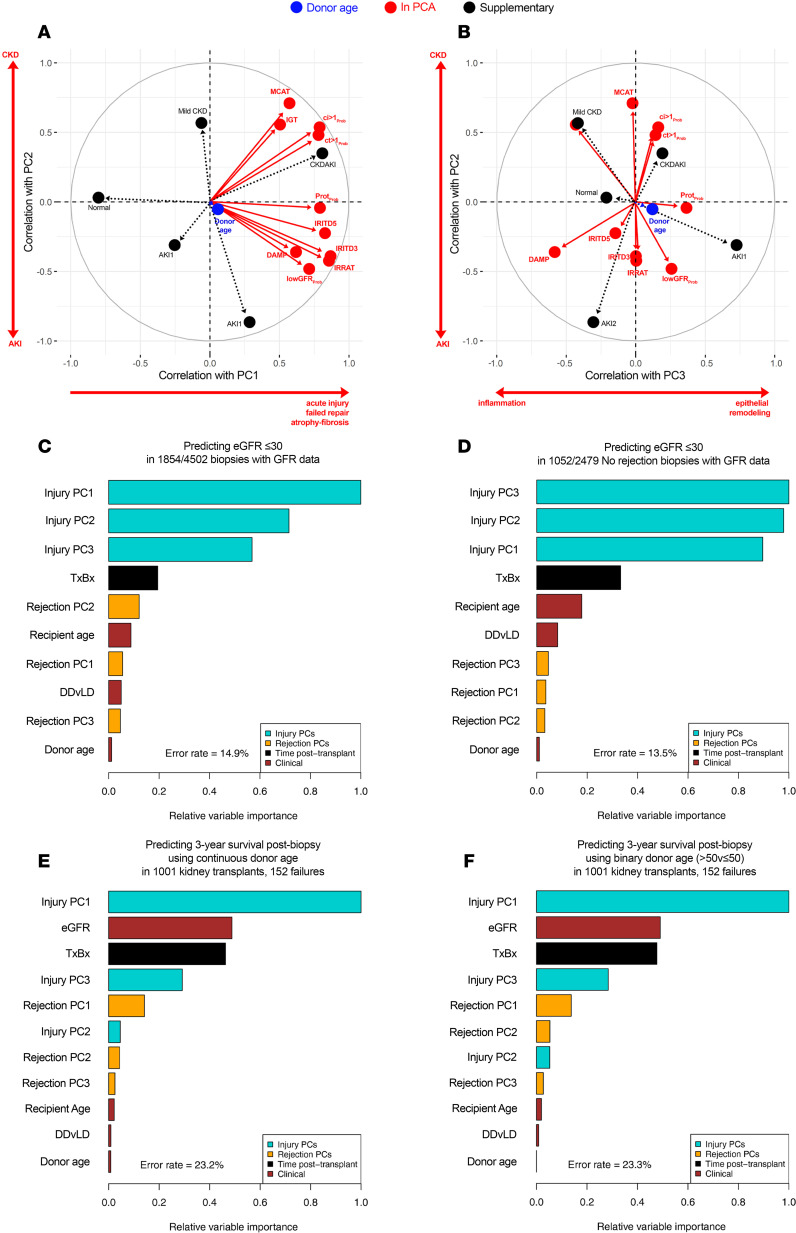
Relationships between injury and rejection molecular features as well as donor age, recipient age, and time after transplant with injury PC1/2/3, and variable importance in predicting eGFR and 3-year postbiopsy graft survival. Factor maps in the *N* = 4,502 kidney transplant biopsy population showing the correlations between the input variables (red circles) and the principal components in the *N* = 4,502 kidney transplant biopsy population. Supplemental variables are shown in black. Donor age was also a supplemental variable and is shown in blue. The correlations between the PCA input variables and the PC scores are shown as factor maps in (**A**) PC2 versus PC1 and (**B**) PC2 versus PC3. **C**–**F** show relative variable importance plots from random survival forest analyses using injury PC1, PC2, and PC3; rejection PC1, PC2, and PC3; donor age; eGFR; recipient age; deceased donor status (DDvLD); and time of biopsy after transplant (TxBx) as predictors for eGFR and 3-year postbiopsy death-censored survival (1 random biopsy per transplant). (**C**) Selected variables predicting eGFR in all 4,502 biopsies; (**D**) predicting eGFR in 2,479 no rejection biopsies; (**E**) predicting 3-year survival after biopsy, including eGFR as an input variable and using donor age as a continuous variable; and (**F**) predicting 3-year survival after biopsy, including eGFR as an input variable and using donor age as a binary variable.

We examined the strength and direction of the correlations of donor age (blue) with direction of injury archetype scores (black) and the injury input variables (red) for the injury PCA (23) (Figure 5, A and B). Donor age had weak relationships to positive injury PC1, negative PC2, and positive PC3, consistent with the correlations reported above but weaker than the other factors shown, suggesting that donor age makes relatively small contributions to total injury changes.

We examined the relative contribution of donor age, recipient age, TxBx, injury PC1/2/3, and rejection PC1/2/3 to predictions of eGFR in a multivariate random forest in all biopsies (Figure 5C) or in no rejection biopsies (Figure 5D). Injury PC1, PC2, and PC3 were dominant in the prediction of eGFR in all biopsies and in no rejection biopsies. Donor age, rejection PC1/2/3, deceased donor status (DDvLD), and recipient age were all relatively unimportant determinants of eGFR.

Similarly, we assessed the relative contribution of donor age and other variables to the prediction of 3-year graft loss after biopsy (Figure 5, E and F). Injury PC1, eGFR, TxBx, and injury PC3 all strongly predicted survival, but donor age, recipient age, rejection PCs, or deceased donor status had only minimal contributions. The findings were similar whether donor age was a continuous variable (Figure 5E) or a dichotomous variable (≥50 vs. <50 years) (Figure 5F).

In univariate or multivariate analyses, neither donor age (*P* = 0.48) nor recipient age (*P* = 0.13) was significantly associated with 3-year postbiopsy survival.

### Relative contribution of donor age to molecular injury and eGFR.

The fact that donor age (univariate or multivariate) had minimal effect on survival was puzzling given the strong statistical associations of donor age with variables that strongly affect survival: IRRAT, injury PC1 and PC3, and eGFR. We examined the relative contribution of donor age to variance in IRRAT (Table 6) and found that donor age made little net contribution to total variance in IRRAT scores (1.4%) or TAL_New4-failed repair scores (2.1%). Similarly, donor age accounts for relatively little of the variance in injury PC1 (0.7%), injury PC3 (3.4%), or eGFR (9%) (data not shown).

Thus, donor age at transplantation had strong interactions with injury measurements and eGFR but little quantitative effect on the observed injury scores, eGFR, or risk of failure.

## Discussion

Given the concerns about donor age (and related risk scores) at the time of the decision to transplant kidneys, we designed the present study to clarify the actual impact of older donor age in kidneys that are transplanted as assessed at the time of biopsy, including kidneys over their entire survival time, day 1 to 45 years after transplant. We examined the correlation between older donor age at transplant (corrected for other predictive variables) and eGFR, injury, and rejection in 4,502 kidney transplant biopsies taken mostly for indications. We focused on the donor age at the time of transplantation but included time after transplant (TxBx) to reflect posttransplant influences, including further aging. The donor age in this population ranged from 1 to 85 years, including 810 biopsies from donors ≥50 years. Donor age correlated with other predictive variables, requiring multivariable analysis (MARS). MARS analysis showed that donor age was the major correlate of eGFR and strongly correlated with increased expression of AKI-induced and failed repair transcripts, as well as injury PC1 and PC3, but had no positive or negative correlations with rejection, which was inversely related to recipient age. As expected (23), AKI-induced and failed repair transcripts behaved very similarly, with peak expression immediately after transplant declining to a plateau after 6 weeks. After time correction, older donor age strongly correlated with CKD-related scores, particularly the CKDAKI archetype score and *CXCL6*. A key finding was that donor age correlations were time-dependent, with maximal effect on AKI/failed repair transcripts and injury PC1 in the intermediate period, peaking about 1 year, not in the immediate after transplant period when these transcripts have peak expression. In contrast, the correlation of donor age with chronicity scores was strong in the early and intermediate periods, similar to its effect on eGFR, suggesting it was established by aging in the donor before transplantation. The genes, gene sets, and pathways increased in donors correlating with older donor age included many previously noted as injury-induced (https://www.ualberta.ca/en/medicine/institutes-centres-groups/atagc/research/gene-lists.html), including the EMT pathway. However, despite significant associations of older age with reduced eGFR and increased molecular injury, the net contribution of donor age to total variance in molecular injury scores and to the prediction of eGFR and 3-year graft survival was surprisingly small, indicating that older donor age has a relatively small impact on the function and outcome of transplanted kidneys compared with other factors.

A surprising finding was that older donor age had relatively little effect on the immediate response to the injuries sustained in the donation-transplantation process, despite strong associations with increased scores at intermediate times. We had expected old kidneys to be more “injurable,” i.e., to find that donor age increased molecular AKI immediately after transplant, but this was not the case. Nevertheless, older donor age was associated with higher AKI/failed repair scores later in the first year after transplant, compatible with failed repair of the transplantation-related injuries. Thus, there was a striking difference between the time of peak expression of AKI/failed repair transcripts (23) and the time course of the effect of donor age on these transcripts.

Molecular chronicity measurements have previously been shown to have low mean values initially (although increased in older donors) and a progressive rise over time. The atrophy-fibrosis classifiers (which correlate strongly with histologic atrophy-fibrosis lesions) and the CKDAKI score and *CXCL6* are lowest in the earliest biopsies but rose steadily — almost linearly — with log time after transplant (23). In contrast, donor age had strong correlations with chronicity measurements in the earliest biopsies and throughout the first year, rising slightly and then falling precipitously to negligible levels at later times as the numerous other sources of injury accumulate. The time course of the effect of donor age on eGFR has some similarities to the chronicity measurements: donor age correlated with low eGFR at early and intermediate times, like chronicity measurements. However, while donor age did not correlate with chronicity scores in late biopsies, some correlation with lower eGFR persisted as expected. We conclude that older donor age correlates with somewhat higher chronicity changes and reduced GFR in the first years after transplant, but there is little evidence of a sustained or progressive impact of donor age as the time after transplant increases.

The correlation of older donor age with increased failed repair transcripts reminds us that failed repair reflects an accumulation of a variety of failed repair cells, some of which share properties with cells that have reached somatic cell senescence, which itself is related to aging (25). Such cells have cytokine secretory potential, may evoke inflammation and increase the risk of progression, and may be vulnerable to strategies similar to senolysis (26). However, the correlations of failed repair transcripts with dysfunction and risk of progression do not necessarily imply causality. Persistence of failed repair cells may also indicate failure to restore the cell mass necessary to maintain normal nephron integrity and function, and the failure to restore nephron cell mass may be the reason for impaired function and survival.

The epithelial remodeling process represented by the positive PC3 score is mildly increased in the early biopsies (23) and correlates with older donor age at all times in the present studies, more strongly in the early period but also in the late period. Indeed, the only sustained correlations of donor age in the late biopsies are with increased PC3 and reduced eGFR. However, the overall contribution of injury PC3 on reduced eGFR and reduced survival in this and previously analyzed cohorts (23) seems to be relatively small, similar to that of donor age.

Two interesting incidental observations in this study are that older kidneys do not get more rejection than younger kidneys and that deceased donor kidneys do not get more rejection than live donor kidneys. Deceased and live donors had similar donor age and time after transplant. Deceased donor kidneys had lower eGFRs and more molecular injury (acute injury/failed repair transcripts, injury PC1, and injury PC3) but not molecular rejection. The overall lack of effect of donor age and DD status on the rejection offers reassurance that aging and injury do not affect allorecognition and rejection. The analysis also shows the importance of multivariable analysis: the apparent weak anticorrelation of donor age with rejection in univariate analysis was actually due to recipient age.

These studies of older donor age in kidney transplant biopsies (82% for indications) come with several limitations that must be considered before generalizing to all kidney transplants. First, kidney transplants undergoing indication biopsies are a relevant sample of troubled kidneys but obviously have more pathology than the average unbiopsied renal transplant. Second, the genome-wide microarrays lack the detail that would be achieved with deep RNA sequencing, but they have the advantage of high standardization in measurements that allows normalization of 4,502 biopsy results for the application of machine learning–derived classifiers and archetypal analysis (AA). Third, we do not have biopsies in these kidneys at the time of donation before transplantation for comparison. (We are performing such studies in a set of kidneys biopsied at the time of removal for transplantation, and injury measurements in the pretransplantation biopsies of old donor kidneys look similar to the changes recorded here in the early after transplant biopsies; data not shown.) Fourth, because we lack long-term postbiopsy data beyond 3 years after biopsy, the long-term effects of donor aging beyond 3 years after biopsy could be underestimated. Finally, we were unable to study the influence of prolonged cold ischemic time, which can be increased by the difficulty in placing older kidneys from older donors.

Our emerging model for kidney transplant injury is that brain death, ICU maintenance, preservation, and transplantation evoke an injury-repair response that is manifest rapidly in the transcriptome (e.g., IRRAT, IRITD3, TAL_New4) and superimposed on existing aging-related changes. Older donor age does not increase the early acute injury changes, which largely resolve over the first 4–6 weeks after injury but does increase the persistence of failed repair cells. Older kidneys come to transplantation with aging-related changes: lower GFR, nephron loss, increased chronicity scores (the atrophy-fibrosis classifiers, CKDAKI, and chemokine *CXCL6*) (38), and EMT-related changes (40). But in the biopsied population represented here, the net contribution of donor age to these changes is relatively small compared with other factors such as rejection, infection, and drug toxicity that are not increased by older donor age, resulting in donor age having a relatively small net effect on eGFR at time of biopsy or on survival 3 years after biopsy. We found no evidence that older donor age automatically imposes accelerated progression to failure in the 4,502 biopsies studied here.

Our findings provide some context for the discussions of transplanting more kidneys from older donors. Older kidneys bring their existing aging changes and a strong tendency toward failed repair but otherwise can be stable in the long term. Older donor age brings with it mild CKD and CKDAKI molecular changes and increases failed repair gene sets, which, per se, correlate with increased risk of failure in kidney transplants (32, 35, 41–43), but the net contribution of donor age at transplantation to these changes is relatively small, at least as seen in the biopsy population. The weakness of the direct relationships of donor age to either eGFR or survival in random forests suggests that the current beliefs that underlie the high discard rate for older donor kidneys need to be reexamined against the needs of the large number of patients on waiting lists.

## Methods

### Sex as a biological variable.

This study includes both male and female kidney transplant recipients and donors and did not exclude any biopsies based on recipient or donor sex to represent the true transplant population as closely as possible. Similar to the overall registered kidney transplant population (44), we found an overrepresentation of male recipients (62% male recipients). Sex was not considered a biological variable for these analyses, as we focused primarily on population-wide analyses.

### Patient population and data collection.

The biopsy population was selected from 5,086 biopsies reported in earlier papers (36). Samples and their associated phenotype data were collected as part of the Molecular Microscope Diagnostic System (MMDx) Kidney studies (INTERCOMEX, NCT01299168; and Trifecta-Kidney, NCT04239703) (31, 36, 45–51). The study design is shown in Figure 1.

Of the 5,086 biopsies described previously (36), only those with an estimated cortex of >10% were used in these analyses (*N* = 4,502), as in ref. 23, with no other exclusions. Analyses related to donor age used only the 1,913 of 4,502 biopsies that had donor age recorded. Some analyses were done in biopsies classified as molecular no rejection (*N* = 2,479) by the rejection archetypes.

### Biopsy processing.

A portion of a core from each biopsy (mean length 3 mm) was stabilized in RNA*later* (Thermo Fischer Scientific) and shipped to Alberta Transplant Applied Genomics Centre (ATAGC) (http://atagc.med.ualberta.ca) or Kashi Clinical Laboratories at ambient temperature. RNA extraction and processing were performed as previously described (48).

### Gene expression data.

Gene expression was measured using GeneChip PrimeView 219 Human Gene Expression Arrays (Applied Biosystems). These chips generate values for 49,495 probe sets, representing 19,462 unique genes. MMDx reports (available for most of the 4,502 samples) were sent to the participating centers, usually within 2 working days of receiving the biopsy. The molecular scores on the reports were generated automatically based on combinations of select gene expression values processed through molecular classifiers. Pathogenesis-based transcript sets (PBTs) are described on the ATAGC home page (https://www.ualberta.ca/medicine/institutes-centres-groups/atagc/research/gene-lists.html). PBT scores represent “standardized” gene set scores — the mean fold change in expression across all probe sets in the PBT versus the mean expression of the same probe sets in a control group (4 nephrectomy samples).

### PCA and AA.

We recently updated the injury PCA and AA in an expanded biopsy population of 4,502 biopsies (23, 37, 43). We assigned injury PC1, PC2, and PC3 scores and 5 injury archetypes (Supplemental Figure 1, A and B). The input for both the PCA and AA was a 4,502 **×** 10 data matrix made up of 6 injury-related PBT scores (IRRAT, MCAT, IGT, IRITD5, IRITD3, DAMP) and 4 injury classifier scores (Proteinuria [Prot_Prob_ ], low GFR [lowGFR_Prob_], ci-lesion >1 [ci>1_Prob_], and ct-lesion >1 [ct>1_Prob_]). In AA, each biopsy was assigned 5 injury archetype scores (summing to 1.0), reflecting its proximity to each of the 5 archetype centers. Each biopsy was assigned to an AA cluster using its highest archetype score. The injury archetypes were labeled as normal, AKI1, AKI2, mild CKD, and CKDAKI (23, 37).

We also assigned rejection PCA and archetype scores to the 4,502 biopsies (31) (Supplemental Figure 1, C and D), based on the published model in 5,086 biopsies (36). The rejection input was a 5,086 × 7 data matrix made up of 7 classifier scores — those for predicting TCMR, antibody-mediated rejection (ABMR), t-lesion >1, i-lesion >1, cg-lesion >0, ptc-lesion >0, and g-lesion >0. The rejection archetypes were no rejection; TCMR1; TCMR2; early-stage ABMR (EABMR), FABMR, and late-stage ABMR (LABMR); and minor ABMR. Rejection PC1 correlated with overall rejection, PC2 separated TCMR (negative) from ABMR (positive), and PC3 distinguished ABMR stages, EABMR, FABMR, and LABMR, plus a minor ABMR archetype group.

All gene sets, classifiers, PCs, and archetype scores used throughout these analyses are described in Supplemental Table 5.

### Survival analysis.

Survival was defined as death-censored graft loss by 3 years after biopsy, selecting 1 random biopsy per transplant for analysis from samples that had donor age data (*N* = 1,001, 152 failures). We used the default parameter settings in the function ‘rfsrc()’ (52) other than importance = “permute,” ntree=5000, nsplit=1, and na.action=”na.impute.” Error rates were defined as 1.0 – the C-statistic, measured in the out-of-bag samples. Relative variable importance used rfsrc’s permutation method.

### Statistics.

All analyses were performed using the R computing language (53) version 4.4.1. Restricted cubic splines with 3 knots were generated using the rms package (54). Differential gene expression was analyzed using the limma package (55). GSEA used the fgsea package (56). MARS was used to model and visualize the relationships between variables. MARS is a nonparametric regression method that models the relationship between predictors and a response by automatically selecting and combining piecewise linear splines at adaptive knots. It is useful for modelling nonlinear and interaction effects. We used the implementation in the R earth package (57) and assessed variable importance within cross-validation via the caret package (58). MARS partial dependence plots correspond to the final models with optimal parameter values, not the individual cross-validated models. Comparison of means used 2-tailed Wilcoxon’s tests. Estimates of the proportion of variance explained by the predictors in linear regression used the relaimpo package (59), PCA used the FactoMineR package (60), and AA used the archetypes package (61). Random forest classification and survival analysis used the randomForestSRC package (52).

### Study approval.

Histology, diagnoses, clinical data, and donor-specific antibody (DSA) status were assigned by the local center’s standard of care following Banff guidelines, as approved by institutional review boards (see list in Supplemental Data). Data were transmitted from the local centers to the ATAGC via electronic or printed forms.

### Data availability.

CEL files are available on the Gene Expression Omnibus, accession GSE275126. Data in the manuscript are included in the Supporting Data Values file.

## Author contributions

PFH was the principal investigator, edited and reviewed the manuscript, and was responsible for data interpretation and study design. KMT was responsible for editing and reviewing the manuscript. M Mackova was responsible for biopsy processing and reviewing the manuscript. JC was responsible for data analysis and reviewing the manuscript. EA, TA, SA, MA, RB, MB, CB, GB, DB, JB, KB, AC, KVC, MC, DDF, DDM, ADS, AD, LD, MD, GE, FE, RF, IF, JF, John Gill, Jagbir Gill, M Glyda, SG, M Gryczman, GG, P Hruba, P Hughes, AJ, ZJ, LK, MK, SK, NK, JK, JL, RM, AM, JM, M Miglinas, TM, M Myślak, BN, AP, APP, MP, GP, EP, SRB, TS, S Shin, S Shojai, MS, JS, KSJ, AS, ZVH, OV, KVS, MRW, AW, ZW, and ZZ were responsible for biopsy sample collection and reviewing the manuscript.

## Conflict of interest

PFH holds shares in Transcriptome Sciences Inc. (TSI), a University of Alberta research company dedicated to developing molecular diagnostics, supported in part by a licensing agreement between TSI and Thermo Fisher Scientific and by a research grant from Natera Inc. PFH is a consultant to Natera Inc., Biogen, and Argenx BV.

## Funding support

- Genome Canada.
- Canada Foundation for Innovation.
- University of Alberta Hospital Foundation.
- Alberta Ministry of Advanced Education.
- Mendez National Institute of Transplantation Foundation.
- Industrial Research Assistance Program.
- One Lambda division of Thermo Fisher Scientific (licensing agreement).

## Supplementary Material

Supplemental data

ICMJE disclosure forms

Supporting data values

## Figures and Tables

**Table 1:**
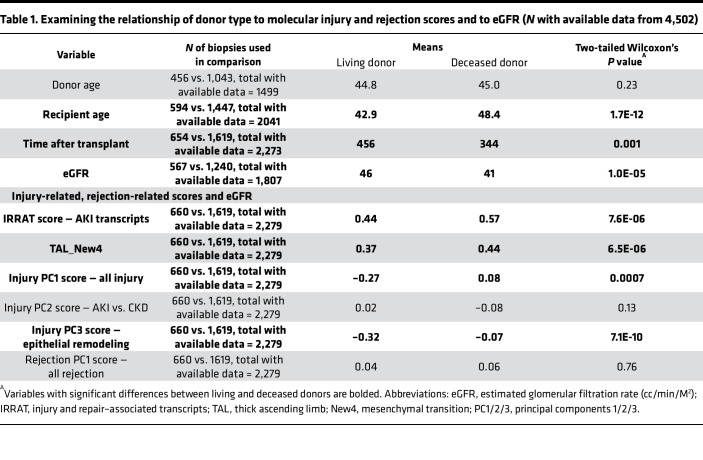
Examining the relationship of donor type to molecular injury and rejection scores and to eGFR (*N* with available data from 4,502)

**Table 2:**
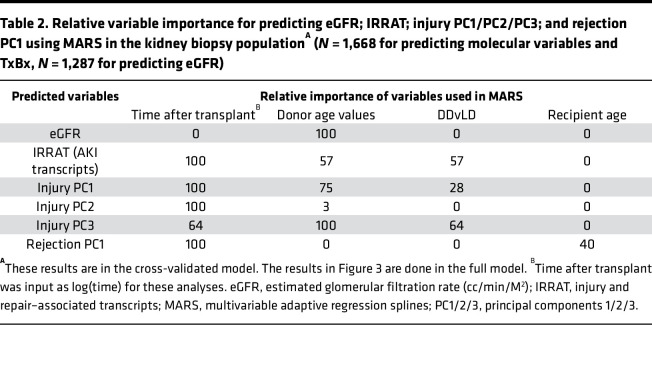
Relative variable importance for predicting eGFR; IRRAT; injury PC1/PC2/PC3; and rejection PC1 using MARS in the kidney biopsy population^A^ (*N* = 1,668 for predicting molecular variables and TxBx, *N* = 1,287 for predicting eGFR)

**Table 3:**
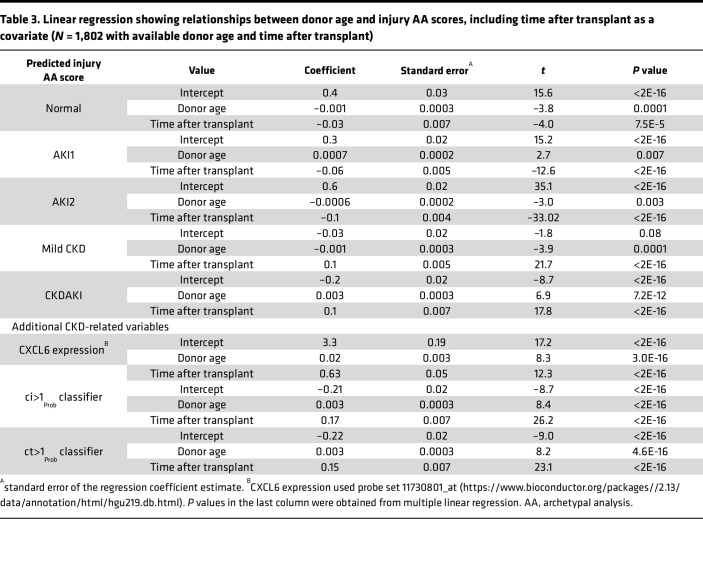
Linear regression showing relationships between donor age and injury AA scores, including time after transplant as a covariate (*N* = 1,802 with available donor age and time after transplant)

**Table 4:**
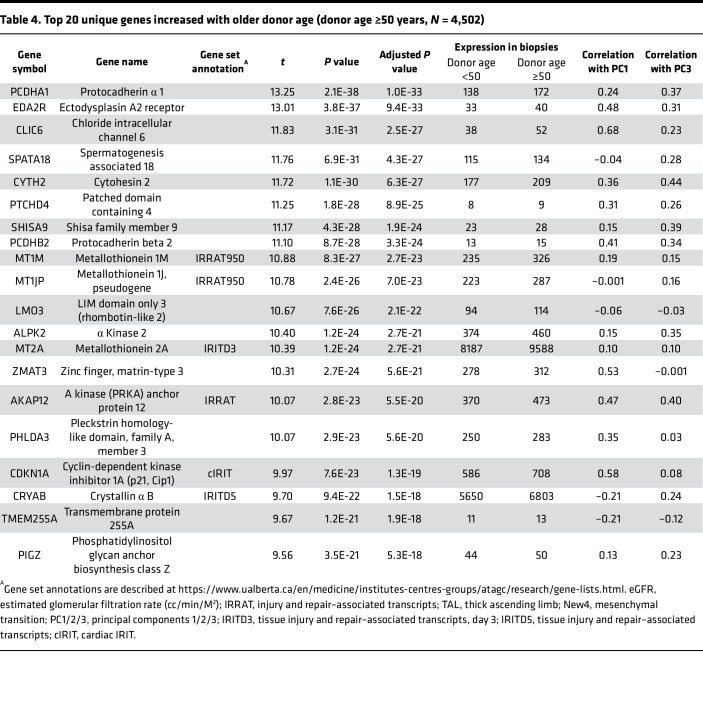
Top 20 unique genes increased with older donor age (donor age ≥50 years, *N* = 4,502)

**Table 5:**
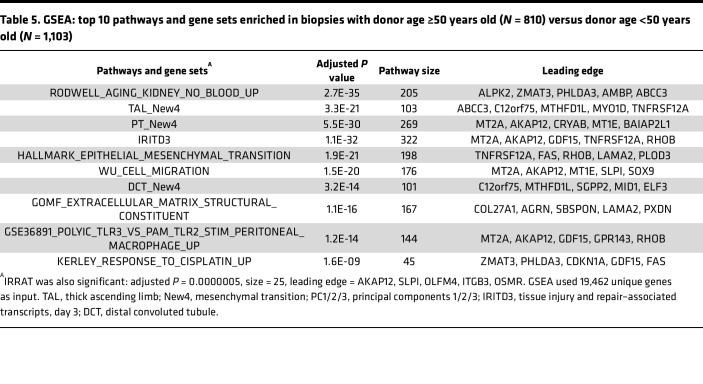
GSEA: top 10 pathways and gene sets enriched in biopsies with donor age ≥50 years old (*N* = 810) versus donor age <50 years old (*N* = 1,103)

**Table 6:**
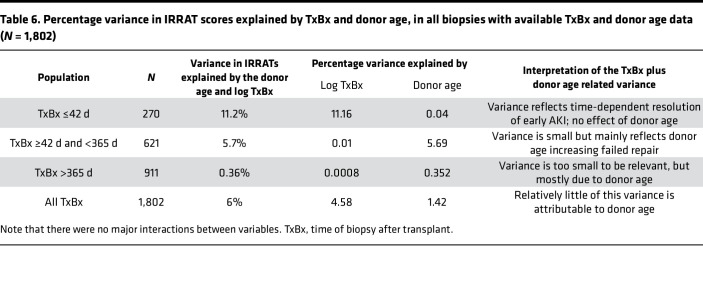
Percentage variance in IRRAT scores explained by TxBx and donor age, in all biopsies with available TxBx and donor age data (*N* = 1,802)

## Version 1 05/07/2026

In-Press Preview

## Version 2 06/22/2026

Electronic publication
