# Supplementary material for: Impact of older donor age in kidney transplants in a biopsy-based observational study
Source: JCI Insight. 2026 May 7;11(12):e199060. doi: 10.1172/jci.insight.199060 (PMC13313539; doi:10.1172/jci.insight.199060)
Supplement: Supplemental data [file jciinsight-11-199060-s269.pdf]

SUPPLEMENTAL MATERIAL

Impact of older donor age in kidney transplants in a biopsy-based observational study

Katelynn Madill-Thomsen[1], Martina Mackova[1], Jessica Chang[1], Enver Akalin[2], Tarek Alhamad[3], Sanjiv Anand[4], Miha Arnol[5], Rajendra Baliga[6], Mirosław Banasik[7], Chris Blosser[8], Georg Böhmig[9], Daniel Brennan[10], Jonathan Bromberg[11], Klemens Budde[12], Andrzej Chamienia[13], Kevin Chow[14], Michał Ciszek[15], Declan de Freitas[16], Dominika Dęborska-Materkowska[15], Alicja Debska-Ślizień[13], Arjang Djamali[17], Leszek Domański[18], Magdalena Durlik[15], Gunilla Einecke[19], Farsad Eskandary[9], Richard Fatica[20], Iman Francis[21], Justyna Fryc[22], John Gill[23], Jagbir Gill[23], Maciej Glyda[24], Sita Gourishankar[25], Marta Gryczman[18], Gaurav Gupta[26], Petra Hruba[27], Peter Hughes[14], Arskarapurk Jittirat[28], Zeljka Jurekovic[29], Layla Kamal[26], Mahmoud Kamel[6], Sam Kant[10], Nika Kojc[30], Joanna Konopa[13], James Lan[23], Roslyn Mannon[31], Arthur Matas[32], Joanna Mazurkiewicz[18], Marius Miglinas[33], Thomas Mueller[34], Marek Myślak[18], Beata Naumnik[22], Anita Patel[21], Agnieszka Perkowska-Ptasińska[15], Michael Picton[16], Grzegorz Piecha[37], Emillio Poggio[20], Silvie Rajnochová Bloudíčkova[27], Thomas Schachtner[34], Sung Shin[35], Soroush Shojai[25], Majid Sikosana[25], Janka Slatinská[27], Katarzyna Smykal-Jankowiak[24], Ashish Solanki[10], Željka Veceric Haler[30], Ondrej Viklicky[27], Ksenija Vucur[29], Matthew R. Weir[11], Andrzej Wiecek[36], Zbigniew Włodarczyk[37], Ziad Zaky[20], and Philip F Halloran[1, 25,*]

[1]Alberta Transplant Applied Genomics Centre, Edmonton, AB, Canada; [2]Montefiore Medical Center, Bronx, NY, USA; [3]Washington University at St. Louis, St. Louis, MO, USA; [4]Intermountain Transplant Services, Murray, UT, USA; [5]University of Ljubljana, Ljubljana, Slovenia; [6]Tampa General Hospital, Tampa, FL, USA; [7]Medical University of Wrocław, Wrocław. Poland; [8]University of Washington, Seattle, WA, USA; [9]Medical University of Vienna, Vienna, Austria; [10]Johns Hopkins University School of Medicine, Baltimore, MD, USA; [11]University of Maryland, Baltimore, MD, USA; [12]Charite-Medical University of Berlin, Berlin, Germany; [13]Medical University of Gdańsk, Gdańsk, Poland; [14]The Royal Melbourne Hospital, Parkville, Australia; [15]Warsaw Medical University, Warsaw, Poland; [16]Manchester Royal Infirmary, Manchester, UK; [17]University of Wisconsin, Madison, WI, USA; [18]Pomeranian Medical University, Szczecin, Poland; [19]Medical University of Hannover, Hannover, Germany; [20]Cleveland Clinic Foundation, Cleveland, OH, USA; [21]Henry Ford Transplant Institute, Detroit, MI, USA; [22]Medical University in Bialystok, Białystok, Poland; [23]St. Paul's Hospital, Vancouver, BC, Canada; [24]Wojewodzki Hospital, Poznan, Poland; [25]University of Alberta, Edmonton, AB, Canada; [26]Virginia Commonwealth University, Richmond, VA, USA; [27]Institute for Experimental and Clinical Medicine, Prague, Czech Republic; [28]University Hospital Cleveland Medical Center, Cleveland, OH, USA; [29]University Hospital Merkur, Zagreb, Croatia; [30]University of Ljubljana, Ljubljana, Slovenia; [31]University of Alabama at Birmingham, Birmingham, AL, USA; [32]University on Minnesota, Minneapolis, MN, USA; [33]Vilnius University Hospital Santaros Klinikos, Vilnius, Lithuania; [34]University Hospital Zurich, Zurich, Switzerland; [35]University of Ulsan College of Medicine/Assan Medical Center, Seoul, South Korea; [36]Silesian Medical University, Katowice, Poland; [37]University Hospital no. 1, Bydgoszcz, Poland

*corresponding author

INTERCOMEX Study: ClinicalTrials.gov #NCT01299168; **Trifecta-Kidney Study**: ClinicalTrials.gov #NCT04239703

Table of Contents

Supplemental Table 1. Patient demographics and biopsy data in the kidney biopsy 4502 population 3

Supplemental Table 2. Linear relationships between recipient age and rejection archetypal analysis (AA) scores including time posttransplant as a covariate (N=2669 with available recipient age and time posttransplant) 4

Supplemental Table 3. Correlations between injury features and donor age in all kidney biopsies (N=4502) 5

Supplemental Table 4. Correlations between injury features and donor age in kidney biopsies called molecular No rejection (N=2479) 6

Supplemental Table 5. Description of pathogenesis-based transcript sets (PBTs)[A] used in analyses or as input in the injury PCA........ 7

Supplemental Figure 1. Principal component analysis (PCA) and archetypal analysis (AA) in the N=4502 kidney transplant biopsy population. 8

Supplemental Figure 2. Partial dependence plots of MARS (multivariate adaptive regression spline) models predicting TAL_New4 with log(days posttransplant) and donor age as predictors shown in the left and right panels, respectively. 9

Supplement Methods........ 10

Supplemental Table 1. Patient demographics and biopsy data in the kidney biopsy 4502 population[A]

Patient Demographics		All patients (N=3611)	Patients with known donor age≥50 (N=671)	Patients with known donor age<50 (N=894)
**Mean recipient age (range)**		50 (3 – 93)	55 (16 – 81)	46 (10 – 93)
**Recipient gender (% known)**	Male	1129 (62%)	202 (65%)	308 (60%)
	Female	694 (38%)	110 (35%)	204 (40%)
**Ethnicity**	Caucasian	698	154	269
	Black	260	50	102
	Other[B]	504	23	56
	Not available[C]	931	89	90
**Primary Disease**	Diabetic nephropathy	305	52	98
	Hypertension / large vessel disease	140	28	37
	Glomerulonephritis / vasculitis	616	66	152
	Interstitial nephritis / pyelonephritis	34	5	4
	Polycystic kidney disease	188	45	36
	Others	432	67	114
	Unknown etiology	317	50	63
	Not available	361	3	13
Mean, median donor age (range)		43, 45 (1– 85)	59, 57 (50 – 85)	33, 35 (1 – 49)
Donor gender (% known)	Male	551 (50%)	119 (47%)	220 (48%)
	Female	546 (50%)	135 (53%)	242 (52%)
Donor type (% deceased donor transplants)		1461 (72%)	215 (68%)	317 (63%)
**Latest kidney status (% of total)**	Graft failed	208 (16%)	44 (16%)	95 (21%)
	Graft functioning/censored	1078 (84%)	236 (84%)	347 (78%)
	Missing/No graft status available	2325	36	75
	Mean (median) follow-up (functioning grafts) in days	617 (282)	789 (265)	875 (415)
**Biopsy features**		**All biopsies (N=4502)**	**Biopsies with known donor age≥50 (N=810)**	**Biopsies with known donor age<50 (N=1103)**
**Mean, median time of biopsy posttransplant (TxBx) (range)**		1182, 367 days (1 day – 45 years)	839, 270 days (1 – 26 years)	1307, 531 (1 – 32 years)
**Mean, median days to most recent follow-up after biopsy (range)**		858, 564 days (1 – 10.5 years)	821, 437 days (2 – 10.5 years)	918, 630 days (1 – 11 years)
**Early biopsies (< 1 year)**		2093 (49%)	426 (55%)	419 (41%)
**Late biopsies (≥ 1 year)**		2208 (51%)	348 (45%)	609 (59%)
**Indication for biopsy**	For Cause	2047 (82%)	642 (80%)	886 (82%)
	Surveillance	439 (18%)	157 (20%)	195 (18%)
	Not recorded	2016	11	22
**Local DSA status at biopsy, if tested**	DSA-positive	842 (48%)	251 (44%)	375 (49%)
	DSA-negative	919 (52%)	314 (56%)	391 (51%)
	Not done	2741 (61%)	245	337
**Histology diagnoses**	ABMR	397 (9%)	107 (13%)	171 (16%)
	AKI	114 (3%)	60 (7%)	40 (4%)
	BK	47 (1%)	19 (2%)	19 (2%)
	Borderline	133 (3%)	50 (6%)	55 (5%)
	IFTA	197 (4%)	89 (11%)	71 (6%)
	Mixed	82 (2%)	20 (2%)	46 (4%)
	NOMOA	383 (9%)	114 (14%)	181 (16%)
	pABMR	116 (3%)	30 (4%)	60 (5%)
	TCMR	152 (3%)	43 (5%)	68 (6%)
	Other	2881 (64%)	278 (34%)	392 (36%)
**MMDx archetypes**	NR	2479 (55%)	505 (62%)	590 (53%)
	TCMR1	231 (5%)	25 (3%)	50 (5%)
	TCMR2	345 (8%)	56 (7%)	96 (9%)
	EABMR	598 (13%)	98 (12%)	148 (13%)
	FABMR	524 (12%)	74 (9%)	148 (13%)
	LABMR	232 (5%)	33 (4%)	55 (5%)
	Minor ABMR	93 (2%)	19 (2%)	16 (1%)

A. Percentages shown are percentage of known data
B. Center specified ethnicity as 'Other'
C. Some centers preferred not to identify ethnicity
Abbreviations: TxBx – time of biopsy posttransplant
Modified from Halloran PF, Chang J, Mackova M, Madill-Thomsen KS, Akalin E, Alhamad T, et al. A cross-sectional study of the role of epithelial cell injury in kidney transplant outcomes. JCI Insight. 2025;10(10):e188658.

Supplemental Table 2. Linear relationships between recipient age and rejection archetypal analysis (AA) scores including time posttransplant as a covariate (N=2669 with available recipient age and time posttransplant)

Rejection AA score	Value	Estimate	Standard error	t	*P* value
No Rejection Score	Intercept	0.52	0.03	15.0	<2E-16
	recipient age	0.002	0.0004	4.6	5.1E-6
	time posttransplant	-0.07	0.008	-8.0	1.7E-15
TCMR1 score	Intercept	0.14	0.01	10.2	<2E-16
	recipient age	-0.001	0.0002	-5.5	3.4E-8
	time posttransplant	-0.02	0.003	-5.1	3.9E-7
TCMR2 score	Intercept	0.11	0.02	6.8	1.4E-11
	recipient age	-0.0006	0.0002	-2.8	0.005
	time posttransplant	-0.0002	0.004	-0.6	0.95
EABMR score	Intercept	0.23	0.02	12.1	<2E-16
	recipient age	0.0005	0.0002	2.2	0.03
	time posttransplant	-0.04	0.005	-8.4	<2E-16
FABMR score	Intercept	0.02	0.02	0.8	0.40
	recipient age	-0.0007	0.0003	-2.5	0.01
	time posttransplant	0.05	0.005	9.4	<2E-16
LABMR score	Intercept	-0.07	0.01	-5.1	3.6E-7
	recipient age	-0.0003	0.0002	-1.7	0.09
	time posttransplant	0.07	0.0003	22.1	<2E-16
Minor score	Intercept	0.05	0.01	5.6	2.3E-8
	recipient age	-3.1E-5	0.0001	-0.3	0.8
	time posttransplant	3.2E-3	0.002	1.3	0.18

Supplemental Table 3. Correlations between injury features and donor age in all kidney biopsies (N=4502)

**Scores for gene sets, classifiers, and injury archetypes**[A]		**All biopsies (N=4502)**		**Biopsies ≤42 days (N=639)**		**Biopsies >42 days and ≤1y (N=1504)**		**Biopsies >1 year (N=2158)**	
		**Spearman correlation coefficient**	***P* value**	**Spearman correlation coefficient**	***P* value**	**Spearman correlation coefficient**	***P* value**	**Spearman correlation coefficient**	***P* value**
Recent injury/failed repair gene sets	IRITD3	**0.12**	**6E-08**	0.01	0.88	**0.22**	**6E-08**	0.05	0.11
	IRRAT	**0.14**	**1E-09**	0.02	0.70	**0.22**	**3.5E-08**	0.06	0.06
	TAL_New4	**0.15**	**8E-11**	0.12	0.05	**0.24**	**1.7E-09**	0.08	0.02
Mitosis	MKI67[B]	-0.06	0.007	-0.15	0.01	-0.12	**0.003**	-0.07	0.05
Epithelial dedifferentiation gene set	KT1	-0.06	0.007	0.02	0.79	**-0.13**	**0.001**	-0.02	0.63
	KT2	-0.09	4E-05	-0.05	0.43	**-0.16**	**7.2E-05**	-0.05	0.16
Chronic injury (atrophy-fibrosis) related scores	IGT	**-0.12**	**7E-08**	0.004	0.94	0.05	0.3	**-0.12**	**0.0003**
	CXCL6	**0.16**	**2E-12**	**0.25**	**4E-05**	**0.29**	**4E-13**	**0.13**	**5E-05**
	Classifier ci>1$_{Prob}$	**0.12**	**1E-07**	**0.37**	**2E-10**	**0.33**	**5.2E-17**	**0.13**	**0.0001**
	Classifier ct>1$_{Prob}$	**0.14**	**8E-10**	**0.37**	**2E-10**	**0.34**	**4.3E-18**	**0.14**	**3E-05**
Injury classifiers	Classifier lowGFR$_{Prob}$	**0.18**	**5E-16**	0.10	0.10	**0.26**	**8.6E-11**	**0.11**	**0.001**
	Classifier Prot$_{Prob}$	0.08	0.0005	0.09	0.15	**0.22**	**3.5E-08**	0.03	0.44
Injury PCA scores	Injury PC1	0.10	1E-05	0.07	0.23	**0.25**	**6.1E-10**	0.05	0.11
	Injury PC2	-0.10	2E-05	0.10	0.09	-0.03	0.5	0.05	0.15
	Injury PC3	**0.20**	**1E-18**	**0.25**	**3E-05**	**0.15**	**0.0002**	**0.22**	**1E-11**
Injury AA scores	Normal	-0.07	0.002	-0.11	0.08	**-0.21**	**2.00E-07**	0.002	0.96
	AKI1	**0.13**	**2.00E-08**	**0.17**	**4.00E-03**	0.07	0.07	0.09	0.01
	AKI2	0.06	0.01	**-0.12**	**0.04**	0.06	0.11	-0.1	0.003
	Mild CKD	**-0.18**	**1.00E-14**	-0.03	0.67	-0.09	0.03	**-0.16**	**2.00E-06**
	CKD AKI	**0.16**	**4.00E-12**	**0.3**	**7.00E-07**	**0.31**	**5.00E-15**	**0.15**	**4.00E-06**

[A] PBTs described at https://www.ualberta.ca/en/medicine/institutes-centres-groups/atagc/research/gene-lists.html
[B] Mean of MKI67 probesets: 11721145_s_at; 11721143_a_at; 11721146_a_at.
NOTE. Bolding signifies Spearman correlation coefficient>0.10 or <-0.10 and *P*<0.01.

Supplemental Table 4. Correlations between injury features and donor age in kidney biopsies called molecular No rejection (N=2479)

**Scores for gene sets, classifiers, and injury archetypes**[A]		**<u>All biopsies</u> (N=2479)**		**<u>Early:</u> Biopsies ≤42 days (N=459)**		**<u>Intermediate:</u> Biopsies >42 days and ≤1y (N=887)**		**<u>Late:</u> Biopsies >1 year (N=996)**	
		**Spearman correlation coefficient**	***P* value**	**Spearman correlation coefficient**	***P* value**	**Spearman correlation coefficient**	***P* value**	**Spearman correlation coefficient**	***P* value**
Recent injury/failed repair gene sets	IRITD3	**0.16**	**4E-08**	0.07	0.32	**0.29**	**2E-08**	0.06	0.19
	IRRAT	**0.19**	**9E-11**	0.11	0.13	**0.30**	**2E-09**	0.09	0.06
	TAL_New4	**0.19**	**1E-10**	**0.17**	**0.02**	**0.30**	**5E-09**	**0.10**	**0.04**
Mitosis	MKI67[B]	0.0003	0.99	-0.05	0.48	**-0.12**	**0.02**	0.01	0.86
Epithelial dedifferentiation gene set	KT1	**-0.13**	**2E-05**	-0.08	0.29	**-0.19**	**2E-04**	-0.07	0.16
	KT2	**-0.15**	**5E-07**	-0.13	0.08	**-0.20**	**8E-05**	**-0.10**	**0.03**
Chronic injury (atrophy-fibrosis) related scores	IGT	-0.07	0.03	0.02	0.80	**0.14**	**0.01**	-0.08	0.08
	CXCL6	**0.18**	**2E-09**	**0.30**	**2E-05**	**0.34**	**8E-12**	0.09	0.06
	Classifier ci>1$_{Prob}$	**0.15**	**4E-07**	**0.39**	**1E-08**	**0.41**	**3E-16**	**0.11**	**0.02**
	Classifier ct>1$_{Prob}$	**0.16**	**1E-07**	**0.37**	**1E-07**	**0.39**	**2E-15**	**0.12**	**0.01**
Injury classifiers	Classifier lowGFR$_{Prob}$	**0.22**	**3E-13**	**0.13**	**7E-02**	**0.30**	**4E-09**	**0.13**	**6E-03**
	Classifier Prot$_{Prob}$	**0.13**	**2E-05**	**0.15**	**0.03**	**0.24**	**3E-06**	0.04	0.44
Injury PCA scores	Injury PC1	**0.15**	**4E-07**	0.13	0.07	**0.33**	**3E-11**	0.07	0.16
	Injury PC2	**-0.10**	**6E-04**	0.05	0.48	-0.01	0.90	0.03	0.55
	Injury PC3	**0.19**	**8E-11**	**0.26**	**2E-04**	**0.13**	**0.01**	**0.19**	**8E-05**
Injury AA scores	Normal	**-0.14**	**6.00E-06**	**-0.17**	**0.02**	**-0.26**	**2.00E-07**	-0.02	0.63
	AKI1	**0.13**	**8.00E-06**	**0.2**	**4.00E-03**	0.06	0.24	0.04	0.38
	AKI2	0.09	0.004	-0.08	0.26	0.07	0.19	-0.08	0.09
	Mild CKD	**-0.13**	**1.00E-05**	-0.04	0.54	-0.02	0.75	**-0.13**	**0.01**
	CKD AKI	**0.17**	**4.00E-08**	**0.29**	**3.00E-05**	**0.35**	**3.00E-12**	**0.12**	**8.00E-03**

[A] PBTs described at https://www.ualberta.ca/en/medicine/institutes-centres-groups/atagc/research/gene-lists.html
[B] Mean of MKI67 probesets: 11721145_s_at; 11721143_a_at; 11721146_a_at.
NOTE. Bolding signifies SCC>0.10 or <-0.10 and p<0.01.

Supplemental Table 5. Description of pathogenesis-based transcript sets (PBTs)[A] used in analyses or as input in the injury PCA

Category	Abbreviation	Description
Recent injury-related	IRITD3[B]	Injury-repair induced, day 3 (IRITD3) (1)
	IRITD5[B]	Injury-repair induced, day 5 (IRITD3) (1)
	IRRAT[B]	Injury-repair associated (IRRAT) (2)
Injury classifiers	lowGFR$_{Prob}$	Classifier predicting GFR ≤30 (3)
	Prot$_{Prob}$	Classifier predicting proteinuria (3)
Late injury (atrophy-fibrosis)-related	IGT[B]	Immunoglobulin transcripts (IGT) (4)
	ci>1$_{Prob}$	Fibrosis score (ci classifier – ci>1 vs ≤1) (5)
	ct>1$_{Prob}$	Tubular atrophy score (ci classifier – ci>1 vs ≤1) (5)
Normal kidney transcripts	KT1	Normal kidney transcripts-set 1 (6)
	KT2	Normal kidney transcripts-set 2 (6)
Macrophage-related	AMAT1	Alternatively activated macrophage (AMAT1) (7)
	QCMAT	Constitutive macrophage (QCMAT) (8)
Damage-associated	DAMPs[B]	Damage associated molecular patterns (9, 10)
Inflammation	MCAT[B]	Mast cell transcripts (11)
"New" injury gene sets	TAL_New4 EMT	Thick ascending limb epithelial mesenchymal transition (12, 13)
	PT_New4	Proximal tubule mesenchymal transition (12-14)
	DCT_New4	Distal convoluted tubule mesenchymal transition (12-14)
Injury PCA scores	PC1	Principal component 1 (14)
	PC2	Principal component 2 (14)
	PC3	Principal component 3 (14)
Injury archetypal score (Injury AAs)	Normal	Normal injury archetypal group (14)
	AKI1	Acute kidney injury 1 archetypal group (14)
	AKI2	Acute kidney injury 2 archetypal group (14)
	Mild CKD	Mild chronic kidney diseases archetypal group (14)
	CKDAKI	Chronic kidney disease with acute kidney injury group (14)

[A] https://www.ualberta.ca/medicine/institutes-centres-groups/atagc/research/gene-lists

[B] PBTs used in injury archetypal analysis

References:

1. Famulski KS, et al. Transcriptome analysis reveals heterogeneity in the injury response of kidney transplants. *Am J Transplant.* 2007;7(11):2483-95.
2. Famulski KS, et al. Molecular phenotypes of acute kidney injury in kidney transplants. *J Am Soc Nephrol.* 2012;23(5):948-58.
3. Einecke G, et al. Factors associated with kidney graft survival in pure antibody-mediated rejection at the time of indication biopsy: Importance of parenchymal injury but not disease activity. *Am J Transplant.* 2021;21(4):1391-401.
4. Einecke G, et al. Expression of B cell and immunoglobulin transcripts is a feature of inflammation in late allografts. *Am J Transplant.* 2008;8(7):1434-43.
5. Halloran PF, et al. Molecular phenotype of kidney transplant indication biopsies with inflammation in scarred areas. *Am J Transplant.* 2019;19(5):1356-70.
6. Einecke G, et al. Early loss of renal transcripts in kidney allografts: relationship to the development of histologic lesions and alloimmune effector mechanisms. *Am J Transplant.* 2007;7(5):1121-30.
7. Famulski KS, et al. Interferon-gamma and donor MHC class I control alternative macrophage activation and activin expression in rejecting kidney allografts: a shift in the Th1-Th2 paradigm. *Am J Transplant.* 2008;8(3):547-56.
8. Famulski KS, et al. Defining the canonical form of T-cell-mediated rejection in human kidney transplants. *Am J Transplant.* 2010;10(4):810-20.
9. Land WG, et al. Transplantation and Damage-Associated Molecular Patterns (DAMPs). *Am J Transplant.* 2016;16(12):3338-61.
10. Heil M, and Land WG. Danger signals - damaged-self recognition across the tree of life. *Front Plant Sci.* 2014;5:578.
11. Mengel M, et al. Molecular correlates of scarring in kidney transplants: the emergence of mast cell transcripts. *Am J Transplant.* 2009;9(1):169-78.
12. Hinze C, et al. Single-cell transcriptomics reveals common epithelial response patterns in human acute kidney injury. *Genome Med.* 2022;14(1):103.
13. Hinze C, et al. Epithelial cell states associated with kidney and allograft injury. *Nat Rev Nephrol.* 2024;20(7):447-59.
14. Halloran PF, et al. A cross-sectional study of the role of epithelial cell injury in kidney transplant outcomes. *Jci Insight.* 2025;10(10):e188658.

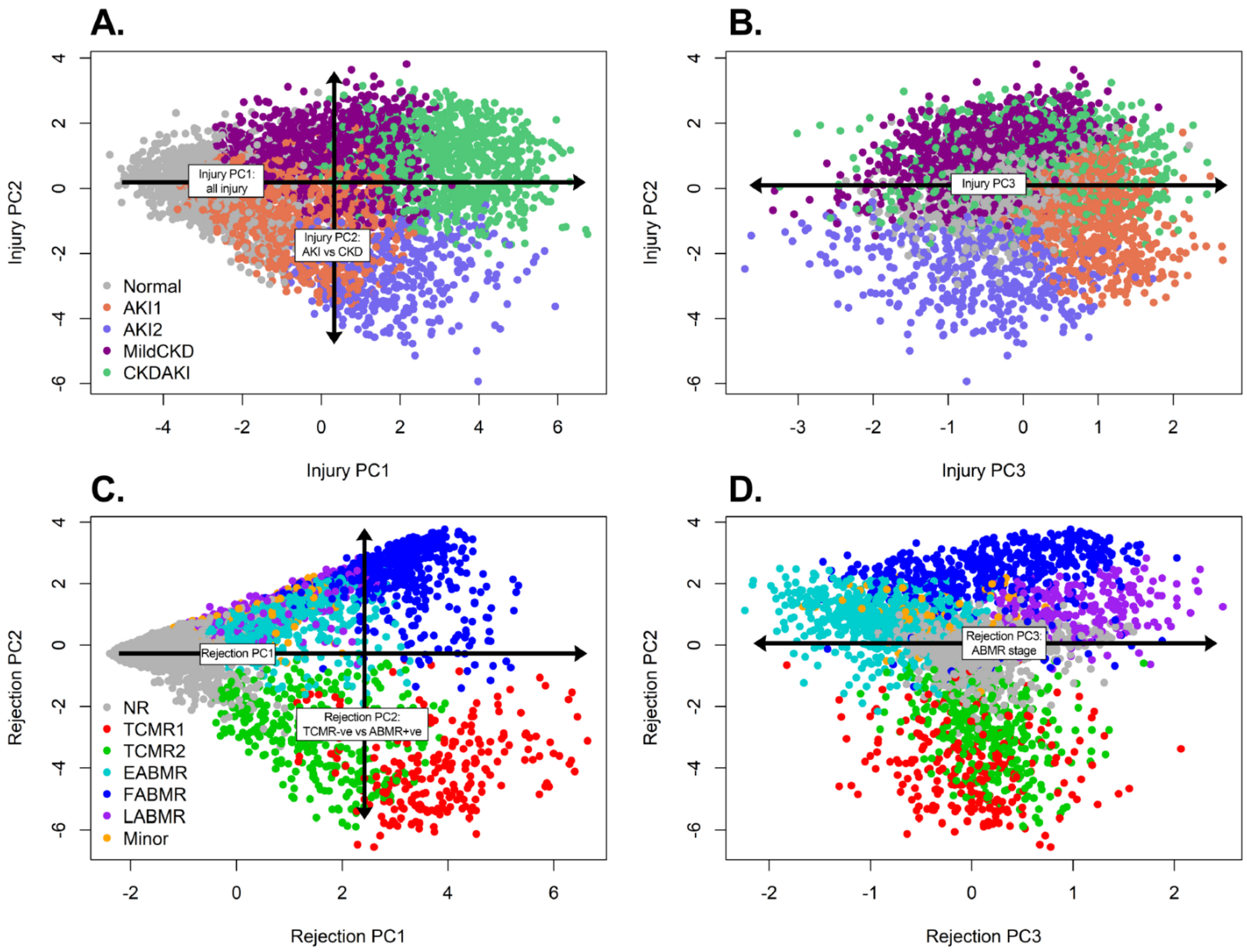

Supplemental Figure 1. Principal component analysis (PCA) and archetypal analysis (AA) in the N=4502 kidney transplant biopsy population. We characterized each biopsy for its molecular injury and rejection in principal component analysis (PCA) and used archetypal analysis (AA) scores to assign groups (23, 36, 47, 48). The 4502 population is distributed using the injury PCAs and colored by the injury archetypal analysis groups, showing A) PC2 vs. PC1, and B) PC2 vs. PC3. Injury principal component 1 (PC1) captures acute injury, failed repair, and chronic kidney disease (CKD) changes; injury PC2 captures time-dependent changes; and injury PC3 is a novel dimension correlating with epithelial development and remodeling genes. We also distributed the 4502 biopsies using the rejection PCAs, colored by the rejection archetypal analysis groups, showing C) PC2 vs. PC1, D) PC2 vs. PC3. Rejection PC1 captures pan-rejection changes; PC2 separates antibody-mediated rejection (ABMR) from T cell-mediated rejection (TCMR); and PC3 represents the stages of ABMR.

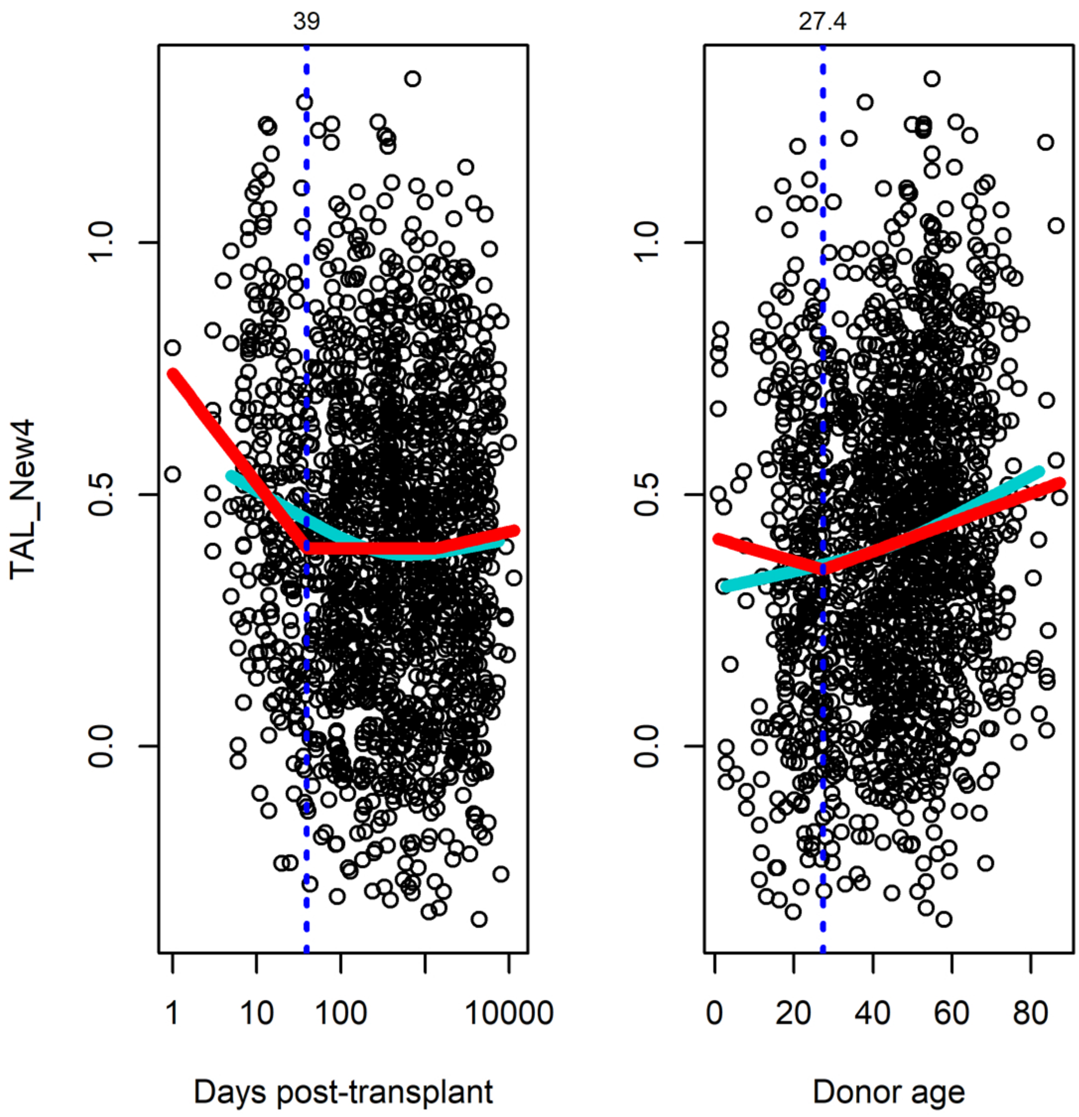

Supplemental Figure 2. Partial dependence plots of MARS (multivariate adaptive regression spline) models predicting TAL_New4 with log(days posttransplant) and donor age as predictors shown in the left and right panels respectively. N = 1802 samples with complete data. Points represent individual biopsies. Red lines are the fitted MARS partial dependence segments. Teal lines represent restricted cubic splines (3 knots). Blue dashed vertical lines indicate segment boundaries selected by MARS, with corresponding values shown above each panel. Predictor variables used in the models were log10(time posttransplant), donor age, recipient age, and living vs deceased donors (0/1). Abbreviations: TAL_New4 – thick ascending limb mesenchymal transition.

Supplemental Methods

IRB approval was obtained from the following institutions: Montefiore Medical Center, Bronx, NY, USA; Washington University at St. Louis, St. Louis, MO, USA; Intermountain Transplant Services, Murray, UT, USA; University of Ljubljana, Ljubljana, Slovenia; Tampa General Hospital, Tampa, FL, USA; Medical University of Wrocław, Wrocław. Poland; University of Washington, Seattle, WA, USA; Medical University of Vienna, Vienna, Austria; Johns Hopkins University School of Medicine, Baltimore, MD, USA; University of Maryland, Baltimore, MD, USA; Charite-Medical University of Berlin, Berlin, Germany; Medical University of Gdańsk, Gdańsk, Poland; The Royal Melbourne Hospital, Parkville, Australia; Warsaw Medical University, Warsaw, Poland; Manchester Royal Infirmary, Manchester, UK; University of Wisconsin, Madison, WI, USA; Pomeranian Medical University, Szczecin, Poland; Medical University of Hannover, Hannover, Germany; Cleveland Clinic Foundation, Cleveland, OH, USA; Henry Ford Transplant Institute, Detroit, MI, USA; Medical University in Bialystok, Białystok, Poland; St. Paul's Hospital, Vancouver, BC, Canada; Wojewodzki Hospital, Poznan, Poland; University of Alberta, Edmonton, AB, Canada; Virginia Commonwealth University, Richmond, VA, USA; Institute for Experimental and Clinical Medicine, Prague, Czech Republic; University Hospital Cleveland Medical Center, Cleveland, OH, USA;University Hospital Merkur, Zagreb, Croatia; University of Ljubljana, Ljubljana, Slovenia; University of Alabama at Birmingham, Birmingham, AL, USA; University on Minnesota, Minneapolis, MN, USA; Vilnius University Hospital Santaros Klinikos, Vilnius, Lithuania; University Hospital Zurich, Zurich, Switzerland; Silesian Medical University, Katowice, Poland; University of Ulsan College of Medicine/Asan Medical Center, Seoul, South Korea; University Hospital no. 1, Bydgoszcz, Poland.
